# Experimental and Analytical Research on Flexural Behavior of Concrete-Filled High-Strength Steel Tubular Members

**DOI:** 10.3390/ma15113790

**Published:** 2022-05-26

**Authors:** Zai-Yu Zhang, Qing Sun, Jia-Qi Wang, Chao Zhao, Bing-Zhen Zhao, Jian-Tao Wang

**Affiliations:** 1 Department of Civil Engineering, Xi’an Jiaotong University, Xi’an 710049, China; zaiyuzhang@163.com (Z.-Y.Z.); sunq@xjtu.edu.cn (Q.S.); jiaqi_cc@163.com (J.-Q.W.); chaozhao@stu.xjtu.edu.cn (C.Z.); 2 Future City Innovation Technology Co., Ltd., Shaanxi Construction Engineering Holding Group, Xi’an 710116, China; tjbhzbz@163.com; 3 SCEGC-XJTU Joint Research Center for Future City Construction and Management Innovation, Xi’an Jiaotong University, Xi’an 710116, China; 4 School of Civil Engineering, Xi’an University of Architecture and Technology, Xi’an 710055, China

**Keywords:** CFHST members, out-of-code *D*/*t* ratios, flexural performance, full-range analysis, bearing capacity model

## Abstract

Using high-strength steel (yield strength *f_y_* ≥ 460 MPa) in concrete-filled steel tubes is expected to provide a superior bearing capacity by achieving light weight and efficient construction, but the existing design limitation on diameter-to-thickness (*D/t*) ratios for concrete-filled high-strength steel tubular (CFHST) members inevitably obstructs its wide application. In this study, aiming at the application of circular CFHST members using Q690 steel (*f_y_* ≥ 690 MPa), a total of 15 CFHST beams were examined using a three-point loading test to investigate the failure mode, bearing capacity and plasticity evolution. Subsequently, finite element models (FEMs) were established to analyze the full-range curves, composite effect, failure mechanism and influences of key parameters including material strengths, *D/t* ratios, and shear-span ratios. A simplified calculation method for bearing capacity was finally proposed and verified. The results indicate that the full-range performance of tested CFHST members with out-of-code *D*/*t* ratios have ductile behavior, though they fail through the mode of steel fracture and concrete cracks in the tension zone as well as through local buckling in the compression zone; out-of-code CFHST members (e.g., *D*/*t* = 120) can perform reasonable composite behavior because of contact pressure larger than 2.5 MPa, where a thin-walled steel tube experiences an arch failure mechanism similar to core concrete at a trussed angle of 45°; the simplified bearing capacity model achieves a mean value of 0.97, and can be accepted as a primary tool to perform structural design and performance evaluation.

## 1. Introduction

**Figure 1:**
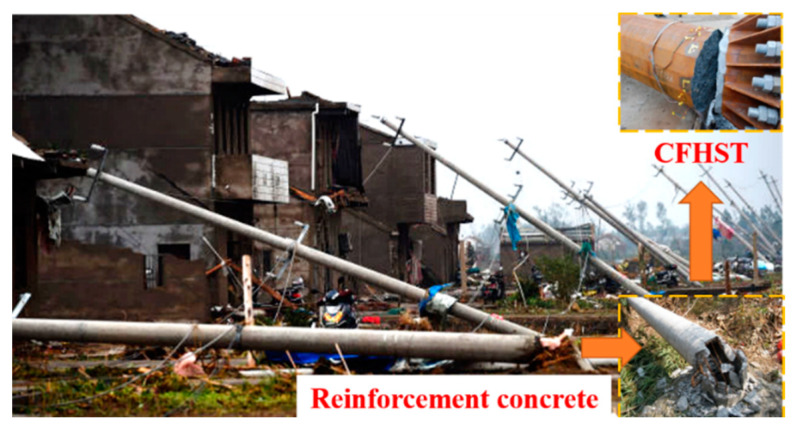
Transmission pole tower collapse.

Due to the interaction behavior between the outer steel tube and core concrete, concrete-filled steel tubular (CFST) members have excellent non-deformability, ductility and bearing capacity for effectively constraining the lateral expansion of concrete and delaying the local buckling of steel tubes [1,2]. In recent years, with the improvement in the production and welding of high-strength (HS) steel (*f_y_* ≥ 460 MPa), CFST using HS steel has been gradually used in structural engineering, e.g., concrete-filled high-strength steel tubular (CFHST) members using Q690 steel (yield strength *f_y_* ≥ 690 MPa) were first applied to transmission towers/poles in China for the purpose of reducing material consumption, weight and cross-sectional area, therefore making a further reduction in lateral wind load and foundation weight [3,4]. However, using HS steel usually triggers a local buckling of thin-walled tubes and fracture damage, although the core concrete still reduces its negative impact [5,6]. For example, experimental and practical investigation demonstrates that the collapse of s CFHST member (e.g., the transmission poles) would display steel fracture and concrete cracking, similar to reinforced concrete structures, leading to building damage, power cuts, and traffic interruption, as shown as Figure 1. Therefore, attention should be urgently paid to composite effects, bearing capacity prediction, and failure mechanisms for the future application of CFHST members.

Many scholars have conducted studies on the flexural performance of CFST members [7,8,9,10,11,12]. For example, Han et al. [13,14] proposed a calculation method to predict ultimate flexural capacity defined by an ultimate state as a steel tube reached 10,000 με. Wang et al. [15] indicated that a conservative prediction for moment-resisting capacity was achieved for Han’s method and the AISC code, meaning that these methods may underestimate the maximum flexural capacity of CFST. Furthermore, a new empirical method based on finite element results was reported by Zand et al. [16], but there is uncertainty in predicting the flexural capacity of HS CFST. Wang et al. [17] investigated six square steel-reinforced CFSTs and four rectangular specimens to evaluate the effect of profiled steel configuration, loading planes, shear span-to-depth (*a*/*D*) ratios and depth-to-width (*D*/*B*) ratios on the flexural performance, and calibrated the accuracy of the existing design method on rectangular steel-reinforced CFST members stipulated in Eurocode 4 (EC4) using experimental results. Shi et al. [18] reported six specimens with various parameters, and proposed a simplified calculation formula. Zhang et al. [19] provided a reliable design method for concrete-filled elliptical steel tubular members by analyzing experimental and numerical results. Moon et al. [20] established a series of numerical models to research the flexural behavior and confinement effect, and proposed a simplified model to predict moment–drift relationships. For CFHST members, Chung et al. [21] revealed that the AISC design method underestimated flexural capacity and stiffness by investigating the flexural capacity of a square CFHST specimen with steel yield strengths of 325 MPa, 555 MPa, and 900 MPa. Xiong et al. [22] investigated the flexural performance of CFST with HS steel (*f_y_* up to 780 MPa) and ultra-high-strength concrete (compressive cylinder strength up to 180 MPa), and proposed a design recommendation by modifying the EC4 design method to predict the flexural capacity. Hanifehzadeh et al. [23] studied a retrofitting system consisting of a UHPC layer enclosed by a thin steel jacket to improve the blast resistance of buildings. Zhou et al. [24] proposed a new type of composite shear wall embedded into high-strength CFST columns and examined the cyclic behavior of the concrete-encased CFST composite wall.

The abovementioned studies provide significant contributions to composite mechanisms and bearing capacity under flexural loading; however, those current design methods still carry an obvious deviation on accurately predicting maximum flexural capacity for CFSTs. For CFHST members, the proposed calculation methods might not be conducive to ductility evaluation and the rational use of bearing capacity. Moreover, the limitations on diameter-to-thickness (*D/t*) ratios specified by EC4, GB 50936-2014 and AISC 360-16 are, respectively, the 90∙235*f_y_*, 177∙235*f_y_* and 0.31∙*E_s_*/*f_y_* [25,26,27,28], for which it might not be reasonable to design CFHST members by neglecting the stronger confinement effect of HS steel, and would restrict the use of HS thin-walled steel tubes, making it hard to achieve reduced material consumption and structural dead weight. Additionally, the study of the composite effect and flexural performance of CFHST members with out-of-code *D*/*t* ratios is still limited. Therefore, it is necessary to investigate the flexural performance of CFHST members with out-of-code *D*/*t* ratios to promote its potential application.

In this study, a total of 15 CFHST specimens with out-of-code *D*/*t* ratios were investigated using a three-point loading test to investigate the failure modes, load–displacement curve, and stress distribution. Subsequently, finite element models (FEMs) were established to analyze the full range of behavior, confinement effect, failure mechanism, and influences of various parameters (e.g., steel strength, concrete compressive strength, *D*/*t* ratio, etc.). Finally, a calculation method on flexural bearing capacity was proposed and verified.

## 2. Experimental Program

### 2.1. Specimen Design

A total of 15 CFHST beams, described in Table 1, were designed to examine the influence of *D*/*t* ratio on flexural performance. Generally, *D*/*t* ratios are not allowed to be greater than 28.5, 56.13, and 84 according to the limitation of EC4, GB 50936-2014, and AISC 360-16, respectively; however, in this study, five groups of tested specimens were, respectively, designed with out-of-code *D*/*t* ratios, including 120, 140, 160, 180, and 200, by maintaining a constant wall thickness of 2 mm. These specimens had a constant span length of 1800 mm, and both ends were supported by a hinge boundary. The regular hexadecagon cross-section was used to closely approximate the circular section due to convenient manufacture. All CFHST members were designed with the combination of HS steel Q690 and concrete C50/60 [26]. The concrete used for the specimen casting is ordinary silicate concrete, which has good workability. It is poured into the steel tube after uniform mixing and then vibrated and compacted by a vibrator many times. After 28 days of normal-temperature curing, a bending test was carried out on the CFST specimens.

### 2.2. Materials

**Figure 2:**
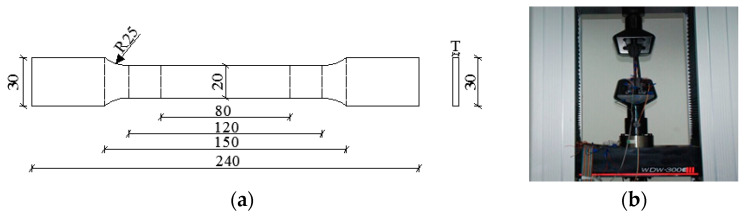
Tensile test of steel coupons. (**a**) Designed size; (**b**) Loading setup.

The mechanical property test of HS Q690 steel was conducted using a tensile testing machine, where the size and loading device were illustrated in Figure 2.

**Figure 3:**
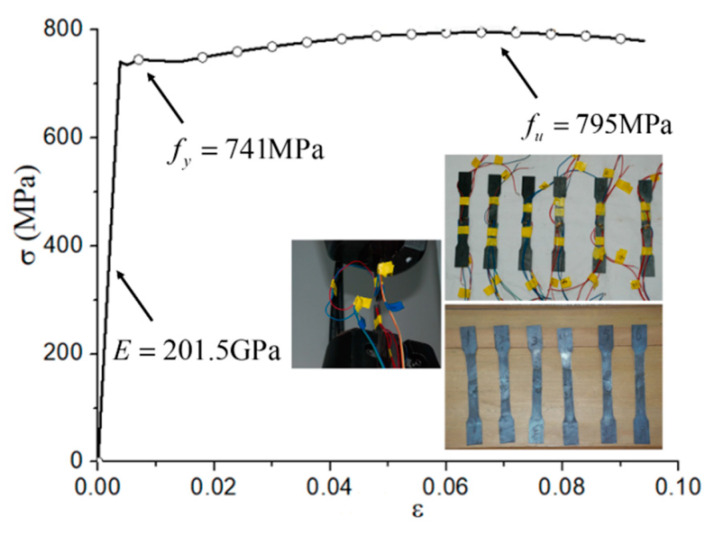
Stress–strain curve of HS steel.

The test was loaded using a displacement controlling mode, and the measured curve of tensile stress versus strain is shown in Figure 3. The average yield strength (*f_y_*) and tensile strength (*f_u_*) of steel coupons were 741 MPa and 795 MPa, respectively. The elasticity modulus (*E*) and Poisson’s ratio were, respectively, 201.5 GPa and 0.24. During the concrete casting, concrete test cubes (150 × 150 × 150 mm) and prisms (150 × 150 × 300 mm) were reserved and cured under the same conditions as the tested CFHST members. The average measured compressive strengths of the cubic specimen (*f_cu_*) was 51.2 MPa, and the elastic modulus of the concrete prism was 31,833 MPa by multiple loading stages according to the method in GB/T 50081-2002 [29]. The maximum aggregate size of concrete is 30 mm, and the mix proportion of concrete infill is shown in Table 2.

### 2.3. Test Setup and Procedure

**Figure 4:**
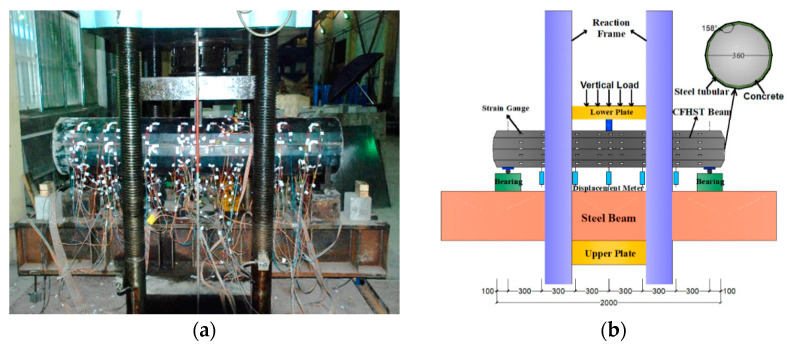
Loading devices. (**a**) Test device; (**b**) Detail drawing of device dimensions.

Considering that the load of the transmission pole tower is only the upper wire and its own weight, the axial compression ratio is relatively small, and the small vertical load has little influence on the maximum bending capacity. Therefore, only the influence of flexural stress on the transmission pole is considered in the test. According to standard GB 4623-2006 for transmission poles, a three-point loading experiment was adopted to examine the flexural performance of CFHST members [30]. Figure 4 shows the loading devices and measuring instruments for the test. To measure the strain of the steel tube at different locations, nine layers of the strain gauges were arranged along the longitudinal axis of the CFHST member, where gauges in every layer were mounted at interval angles of 45° along the circumference. Five strain-displacement meters were arranged at the bottom of the CFHST beam to measure the deflection change. The vertical load controlled by a combination mode of force and displacement was gradually transferred to the mid-span of the specimen through the steel cushion. Before the test, the CFHST beam was preloaded to make the specimen and loading device in full contact, and to check whether the display and data recording were in a normal operation condition. Subsequently, the vertical load was applied and increased by incremental steps of 0.1 *P_cu_*, where *P_cu_* was the predicted target load calculated by numerical models. After reaching 0.6 *P_cu_*, the vertical load was controlled by a displacement loading mode with a continuous speed of 0.2 mm/min. The test was terminated when local buckling and fracture damage to the steel tube, or concrete crushing, obviously occurred.

## 3. Experimental Results and Discussion

### 3.1. Failure Modes

**Figure 5:**
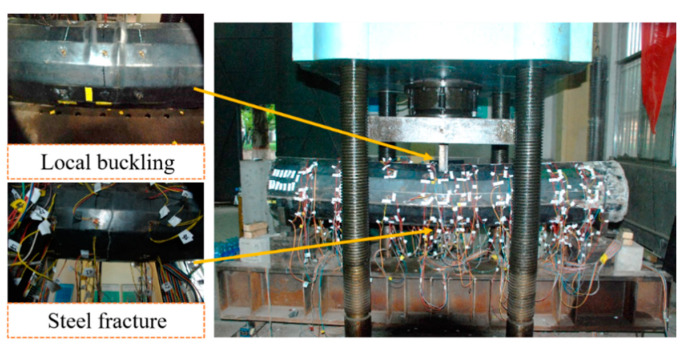
Failure modes of CFHST beams.

The typical failure modes of an out-of-code CFHST member are depicted in Figure 5. It can be concluded that ultimate bearing capacity was generally achieved with an obviously developed steel fracture, and with concrete cracks in the tension zone. Meanwhile, in the compression zone, the HS steel tubes displayed local buckling due to the partial pressure of loading steel cushion. It can be observed that deflection in the middle span increased in a ductile manner to finally form a plastic hinge, therefore leading to bending failure.

### 3.2. Analysis and Discussion

**Figure 6:**
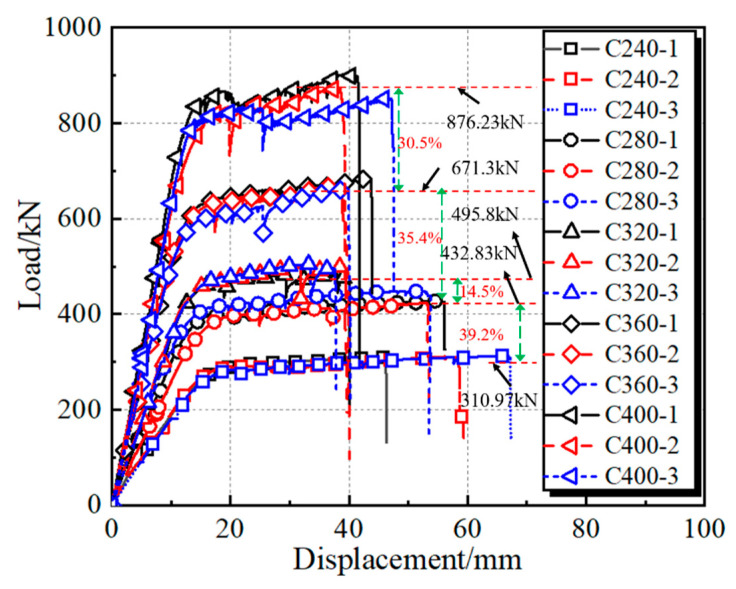
Load–displacement histories of CFHST beams.

Figure 6 shows the curves of load versus displacement at mid-span. It can be seen that the averaged maximum vertical load of groups C240–C400 are, respectively, 310.97, 432.83, 495.80, 671.30 and 876.23 kN, and the stiffness of different curves perform a gradually increasing trend as the diameter of the core concrete increases. Furthermore, the curves of vertical load versus displacement show a slight increasing trend as the specimens enter the plastic stage that displays the obvious ductile capacity due to the hardening of HS steel. However, the curves sharply drop after the steel fracture in the tension zone. Generally, it can be observed that the specimens with small *D*/*t* ratios (Group C240) reach a further failure displacement, which can be ascribed to the higher confinement coefficient *ξ* = *f_y_A_s_*/(*f_ck_A_c_*), where *A_c_* and *A_s_*, respectively, indicate the cross-sectional areas of core concrete and steel tube, and *f_ck_* is the concrete prism strength. To obtain the moment (*M*)–curvature (*ϕ*) relationship of CFHST beams, Equations (1) and (2) were used to derive average curves:

(1) $$\phi =(\varepsilon_{1}-\varepsilon_{2})/(h_{1}-h_{2})$$

(2) M=VL/4

where *ε*_1_ and *ε*_2_ are, respectively, the longitudinal strain at various heights *h*_1_ and *h*_2_ of mid-span cross-section, and *V* is the vertical load.

**Figure 7:**
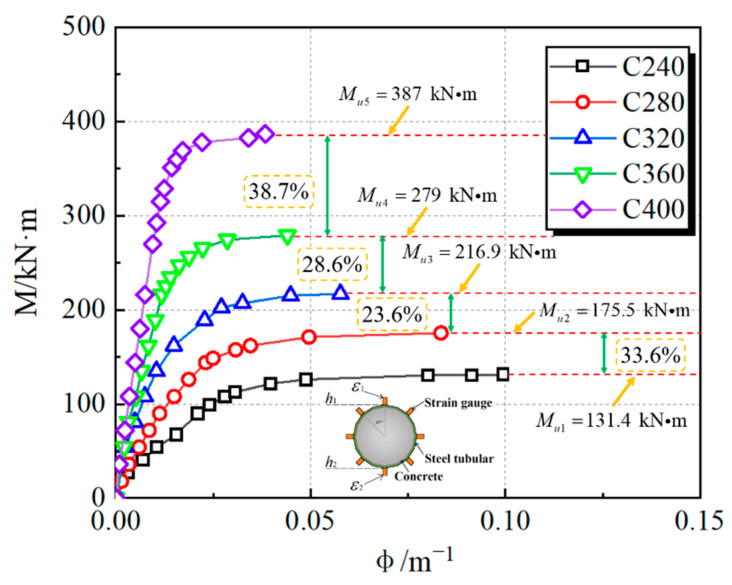
M-ϕ curves of CFHST beams.

Figure 7 depicts the bending moment and section curvature relationships of CFHST beams at different stages, where the averaged maximum bending moment of C240, C280, C320, C360 and C400 are, respectively, 131.4 kN∙m, 175.5 kN∙m, 216.9 kN∙m, 279 kN∙m and 387 kN∙m. As the *D*/*t* ratio increases, the flexural capacity of specimens is, respectively, enhanced by 33.56%, 65.07%, 112.33%, and 194.52%. When the shear-span ratio of specimens is greater than 2, the bending moment plays a dominant role in the failure of the specimens, while the shear force has little effect [31]. Therefore, the effect of shear force on bearing capacity can be ignored.

**Figure 8:**
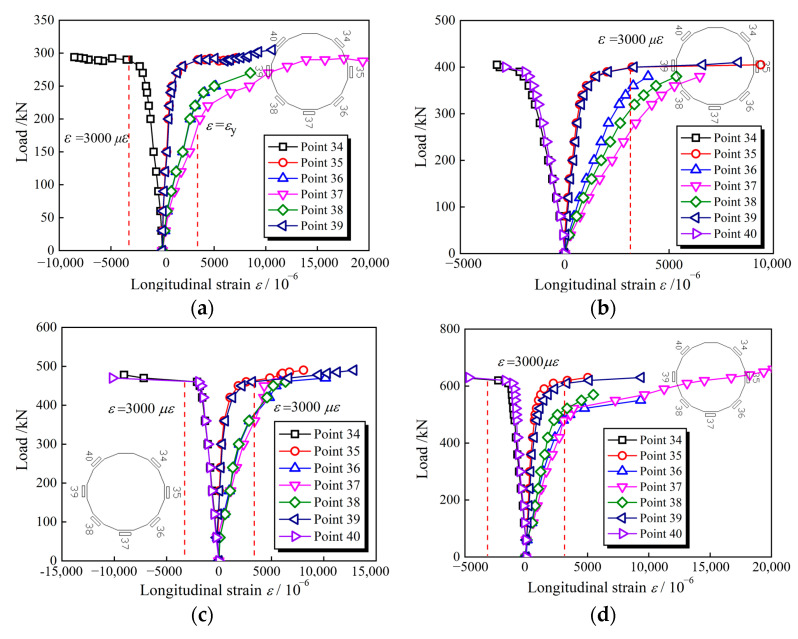

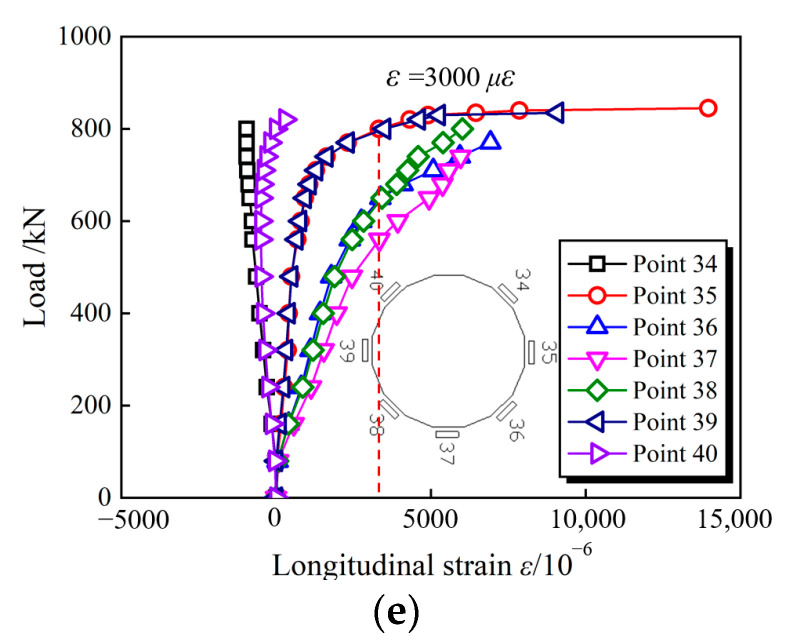
Failure modes of CFHST beams at different stages. (**a**) C240-1; (**b**) C280-1; (**c**) C320-1; (**d**) C360-1; (**e**) C400-1.

Figure 8 presents the measured moment (*M*) versus strain (*ε*) curves for all specimens at the mid-span section. The red dashed line represents the position where the longitudinal strain is equal to 3000 με. It can be concluded that, for the longitudinal strain in the tension zone, it increases faster than in compressive zone, and the longitudinal tensile strain tends first to achieve yield strain compared to compressive strain. Moreover, the longitudinal tensile strain displays a higher anti-deformation capacity, up to 20,000 με (e.g., Point 37 of C240-1, C360-1), reflecting the sufficient plasticity development, although the HS thin-walled steel tubes finally fracture.

**Figure 9:**
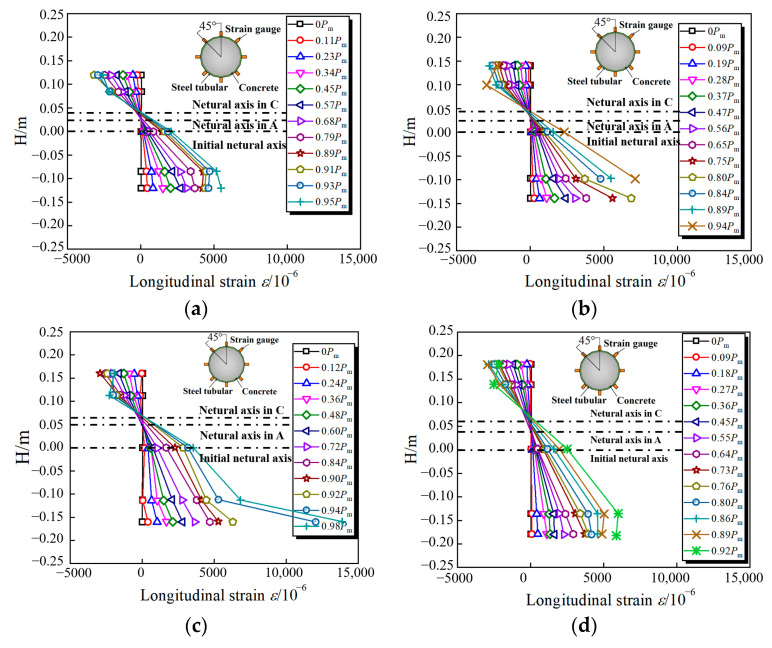

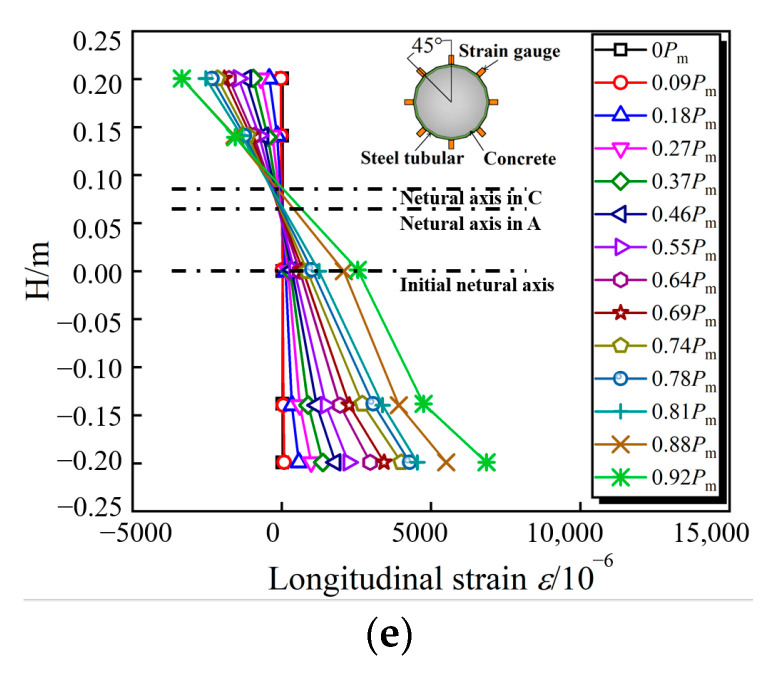
Longitudinal strain distribution at mid-span. (**a**) C240; (**b**) C280; (**c**) C320; (**d**) C360; (**e**) C400.

To investigate the longitudinal strain (*ε*_L_) distribution along the loading process, the strain distributions in the mid-span section were extracted along the section height (*D*). Figure 9 shows the strain value of the CFHST specimen when it is close to peak load. Because the steel suddenly broke when the specimen reached the peak load, the strain value far exceeded the measuring range of the strain gauge, and the corresponding strain value of the peak load point could not be measured. The tensile strain is regarded as a positive sign, and the position of the neutral axis is marked in different stages. At the initial loading stage, the initial neutral axis is on the centroid of the circular section. When the loading force increases, the neutral axis at the mid-span section gradually moves upwards because of the sequential concrete cracks.

Generally, the moving distance is approximately 0.1 *D*. Additionally, the steel tube maintains a minor tension deformation during the elastic stage, but, with continuous loading, the tensile deformation grows quickly after steel begins to yield, bringing about a gradual large sectional curvature. It is noteworthy that strain distribution along the section height approximately performs in a linear trend, revealing that the plane cross-section assumption is justified for CFHST members.

## 4. Numerical Investigation

### 4.1. Finite Element Model

**Figure 10:**
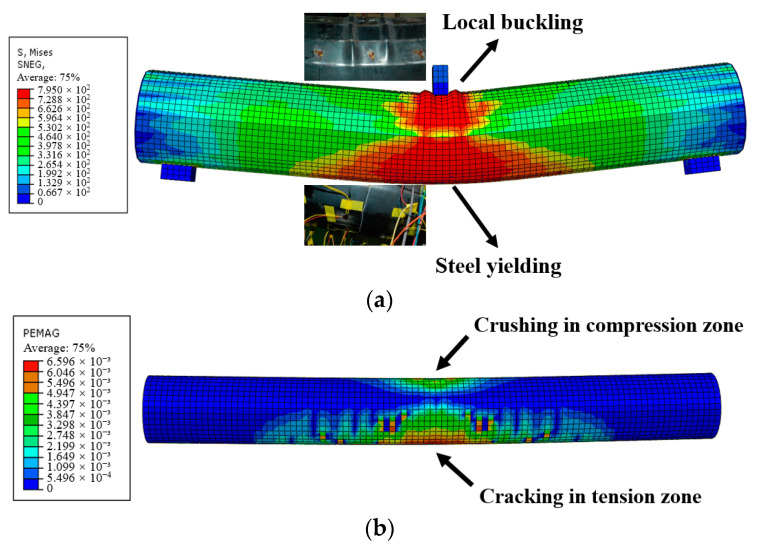
(**a**) CFHST member; (**b**) Core concrete.

To extensively understand and estimate the confinement behavior, failure mechanism, and influences of various parameters, a series of finite element models (FEMs) of the tested specimens were established in ABAQUS software, where the element types of the steel tube and core concrete were, respectively, S4R and C3D8R, as shown in Figure 10. The boundary conditions of FEMs coincided with the test specimens to simulate the hinged supports. To accurately model the interaction behavior, the Coulomb friction model and hard contact were applied in a tangential direction and the normal direction, respectively, based on surface-to-surface contact, for which the friction coefficient was taken to be 0.6 [32]. Meanwhile, the constitutive relationship of HS steel adopted a trilinear model that retains a constant strength while reaching the maximum tensile strength [33]. On the other hand, the concrete damage plastic (CDP) model was used for the core concrete via using the compressive stress–strain relationship of concrete established by Han et al. [34]. The tensile relationship of core concrete was provided by GB 50010-2010 [35]. The constitutive relationship of core concrete can be determined as follows:

(3) $$y=\left\{ \begin{array}{ll} 2\cdot x-x^{2} & \left(x\leq 1\right)\\ \frac{x}{\beta_{0}\cdot {\left(x-1\right)}^{2}+x} & \left(x>1\right) \end{array}\right.$$

(4) $$x=\frac{\varepsilon }{\varepsilon_{0}},\;\;y=\frac{\sigma }{\sigma_{0}}$$

(5) $$\xi =\frac{f_{y}A_{s}}{f_{ck}A_{c}}$$

(6) $$\varepsilon_{0}=\varepsilon_{c}+800\cdot \xi^{0.2}\times 10^{-6}$$

(7) $$\varepsilon_{c}=(1300+12.5\cdot f_{c})\times 10^{-6}$$

(8) $$\beta_{0}={\left(2.36\times 10^{-5}\right)}^{\left[0.25+{\left(\xi -0.5\right)}^{7}\right]}\cdot {\left(f_{c}\right)}^{0.5}\cdot 0.5\geq 0.12$$

where *f_c_* represents the cylinder strength of concrete; *A_c_* and *A_s_*, respectively, indicate the cross-sectional areas of core concrete and steel tube; *ξ* is the confinement coefficient; and *f_ck_* is the concrete prism strength.

### 4.2. Verification of the FEMs

The established FEMs for CFHST members were validated in terms of failure mode and full-range curves of load versus displacement compared to the test results. Figure 10 displays the simulated failure mode, indicating that the established FEMs agreed well with the test specimens, especially in overall bending, local buckling, and steel yielding (Figure 10a), thereafter, Figure 10b illustrates concrete crushing and crack failure in the compression and tension zone. It can be deduced that the failure mode and test phenomenon can be well captured and predicted by FEMs.

**Figure 11:**
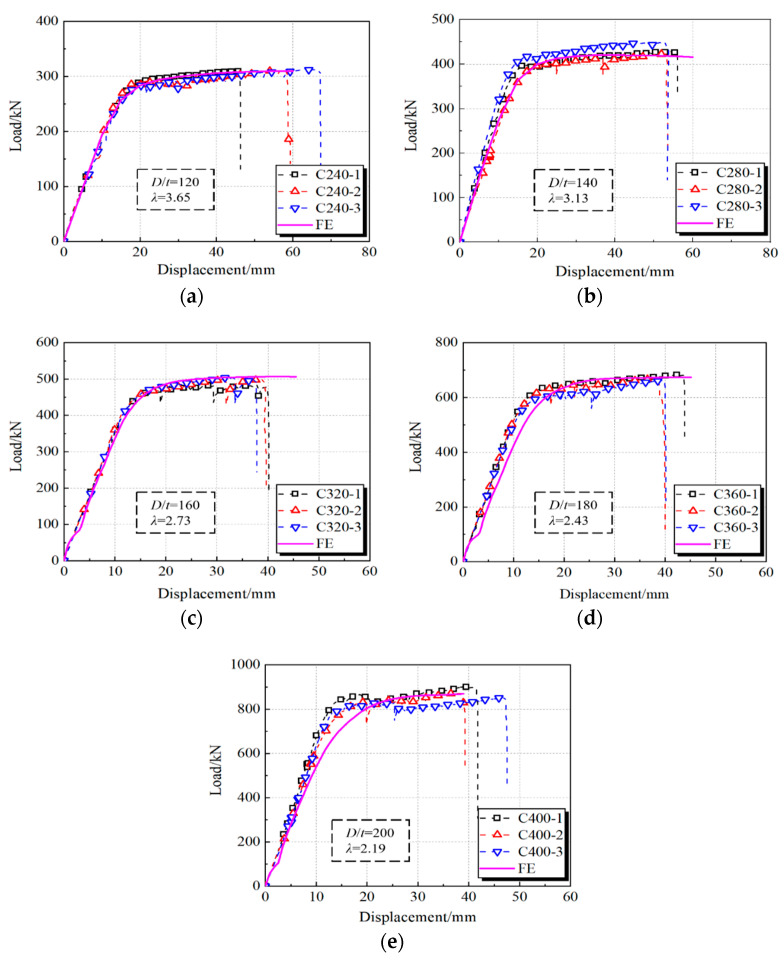
The results of FEM and experiment. (**a**) C240; (**b**) C280; (**c**) C320; (**d**) C360; (**e**) C400.

As for the full-range test curves, as shown in Figure 11, it can be observed that the calculated curves of FEMs coincide well with the characteristics of initial stiffness, ultimate strength, and pre/post-peak behavior compared to the tested members. Certain differences among the comparison results inescapably exist in the buckling extent and bending behavior of the nonlinear stage, which is due to uncertainties in actual material properties and imperfections. To sum up, the developed FEMs in this section reasonably simulate the bending characteristics of CFHST members, and can be accepted for the purposes of further mechanism investigation and parametric study.

### 4.3. Full-Range Analysis on Flexural Performance

**Figure 12:**
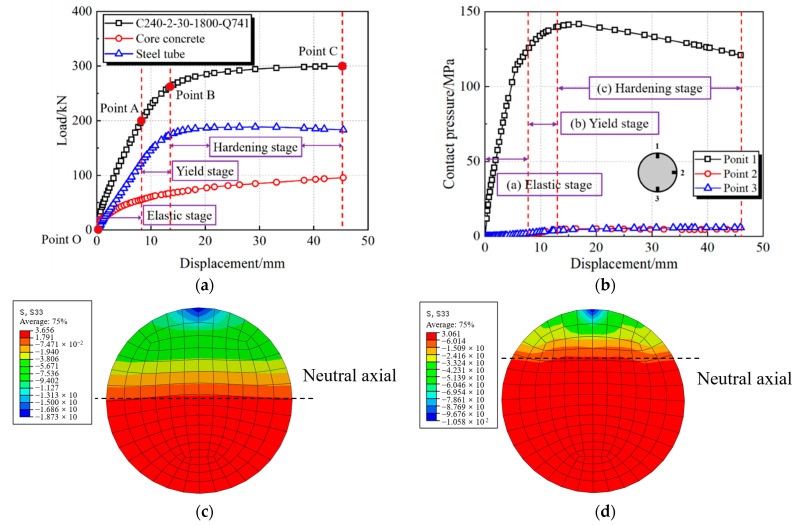
Full-range analysis on a typical CFHST member. (**a**) Load–displacement relation; (**b**) Interaction behavior; (**c**) Elastic stage; (**d**) Yield stage.

One typical CFHST specimen was used for the explanation of full-range bending behavior and composite mechanism, where the diameter (*D*), thickness (*t*), span length (*L*) and compressive cylinder strength of concrete (*f_c_*) are, respectively, 240 mm, 2 mm, 1800 mm and 30 MPa. Additionally, Q741 shows that the yield strength (*f_y_*) of a steel tube is 741 MPa, therefore forming the notation C240-2-30-1800-Q741. As displayed in Figure 12, the load–displacement curve mainly goes through three stages, namely elastic, yield, and hardening stages, and the curves can be divided at three characteristic points. Points A and B are, respectively, defined by the steel tube reaching yield strain in the tension zone and compressive zone, and Point C is determined by the fracture of the steel tube in the tensile zone.

Therefore, the three stages can be summarized as follows.

(1) Stage 1: Elastic stage (Points O–A). In this stage, the CFHST member retains elastic behavior. The load–displacement curve shows a linear growth relationship, and the HS steel tube and core concrete withstand, respectively, 69% and 31% of the specimen flexural capacity at Point A (Figure 12a). In Figure 12b, the contact pressure between the steel tube and core concrete retains a small pressure at Points 2 and 3, since the Poisson ratio of the core concrete is smaller than that of the steel tube, and it can be considered that they work independently. Meanwhile, in Figure 12c, the height of the neutral axis is nearly located at the center line of the cross-section.
(2) Stage 2: Yield stage (Points A–B). The load–displacement curve enters the yield stage, and the loading-carrying proportions of the HS steel tube and core concrete are, respectively, 72% and 28% at Point B. The local buckling of the steel tube in the compressive zone causes the contact pressure to increase slowly at Point 1, and the contact pressures at Points 2 and 3 gradually increase due to the significant expansion deformation of the confined concrete. At the same time, an obvious stress-redistribution phenomenon exists in the compression zone, and the neutral axis increasingly moves upward, owing to the concrete failure in tensile zone, as shown in Figure 12d. During this stage, the steel tube exceeds its corresponding yield strength *f_y_*, and the stress of the core concrete is higher than its cylinder strength *f_c_* due to the confinement effect provided by steel tube.
(3) Stage 3: Hardening stage (Points B–C). The plastic deformation occupies a major part, and the contribution of the steel tube to the flexural capacity generally retains a higher percentage, for which the vertical bearing capacity proportions of the HS steel tube and core concrete are, respectively, 66% and 34% at Point C. A large value of 5.89 MPa can be reached for contact pressure at Point 3 compared to the lower value at Point 2, reflecting a compact interaction between the steel tube and concrete. Although the core concrete suffers from severe damage during this period, the damaged concrete can still effectively resist the ovalization and buckling of the steel tube.

### 4.4. Load-Transfer Mechanism in CFHST Beams

**Figure 13:**
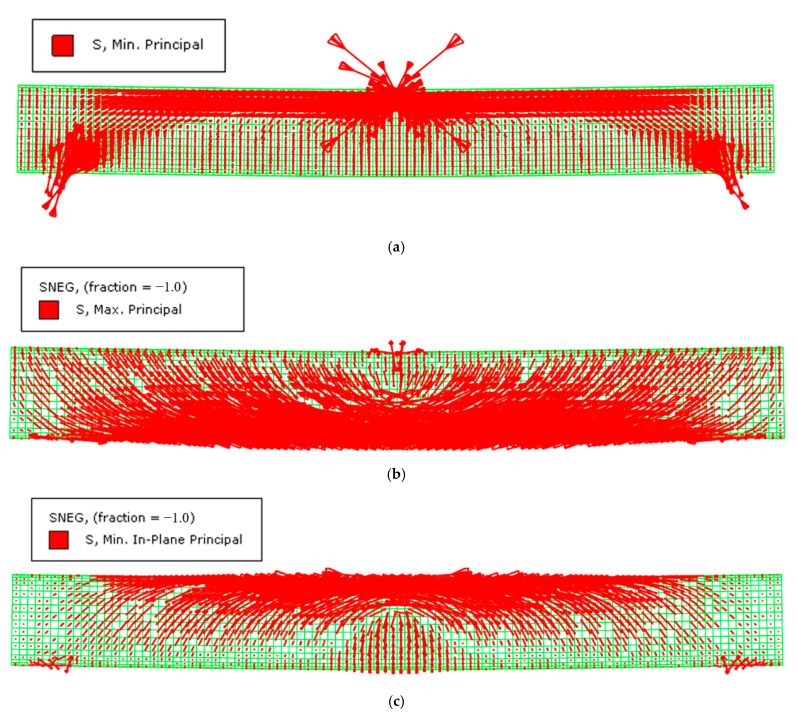
Maximum stress in CFHST beam. (**a**) Maximum compressive stress in concrete; (**b**) Maximum tensile stress in steel tube; (**c**) Maximum compressive stress in steel tube.

In this section, the load-transfer mechanism of the CFHST beam is investigated corresponding to the typical specimen in Section 4.3, and the typical vector symbols of the maximum compressive stress of concrete and the maximum compressive stress and tensile stress of steel tube are depicted in Figure 13, where the arrow represents the stress direction; the arrow length indicates the magnitude of stress; and the arrow density denotes the stress distribution. By analyzing the maximum stress distribution and direction of concrete and steel tube, the load-transfer path of the CFHST member can be revealed.

From Figure 13a, it can be seen that maximum compressive stress is located at the loading points and hinge supports, where an arch pattern is formed to bear the vertical load. The relative depth of the compressive area mainly focuses on one third of the cross-section height. Similar to the core concrete, in Figure 13b the steel tube also experiences an arch mechanism, where the maximum tensile stress transmits at an angle of approximately 45° and vice versa; specifically, the compressive stress of the steel tube in Figure 13c displays a counterpart layout similar to the core concrete in Figure 13a. Therefore, it can be concluded that the failure mode of CFHST members is dominated by bending destruction that first derives from the tension damage of the concrete and steel tube, followed by concrete crushing and local buckling of the steel tube.

### 4.5. Parametric Study

**Figure 14:**
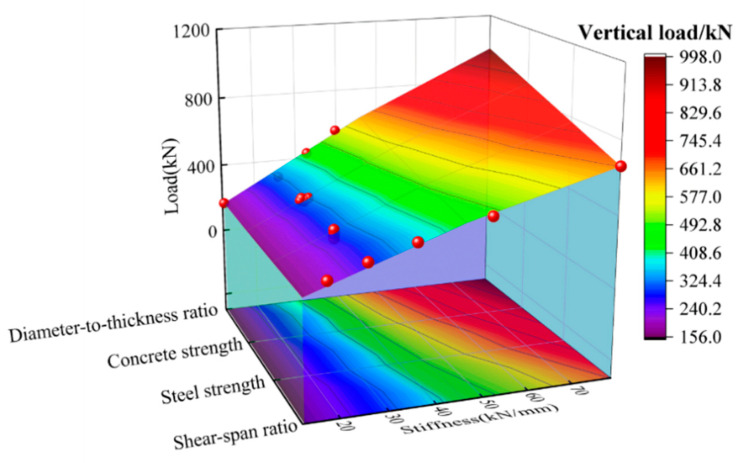
Influences on bearing capacity and stiffness.

**Figure 15:**
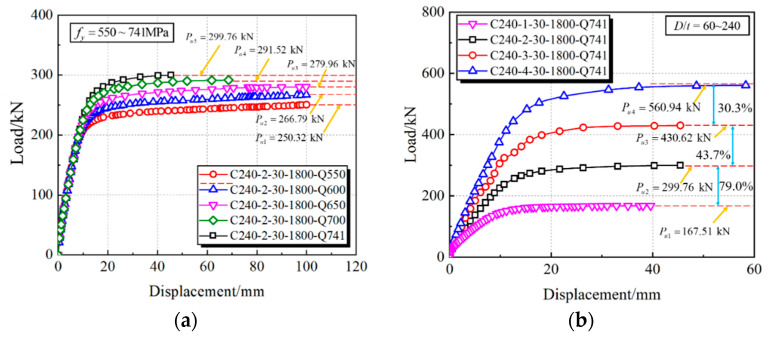

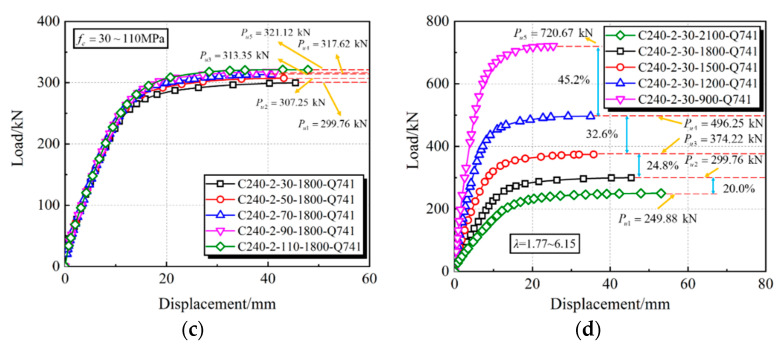
Influence on full-range load–displacement curves. (**a**) Yield strength of steel tube; (**b**) *D*/*t* ratio; (**c**) Compressive strength of core concrete; (**d**) Shear-span ratio.

**Figure 16:**
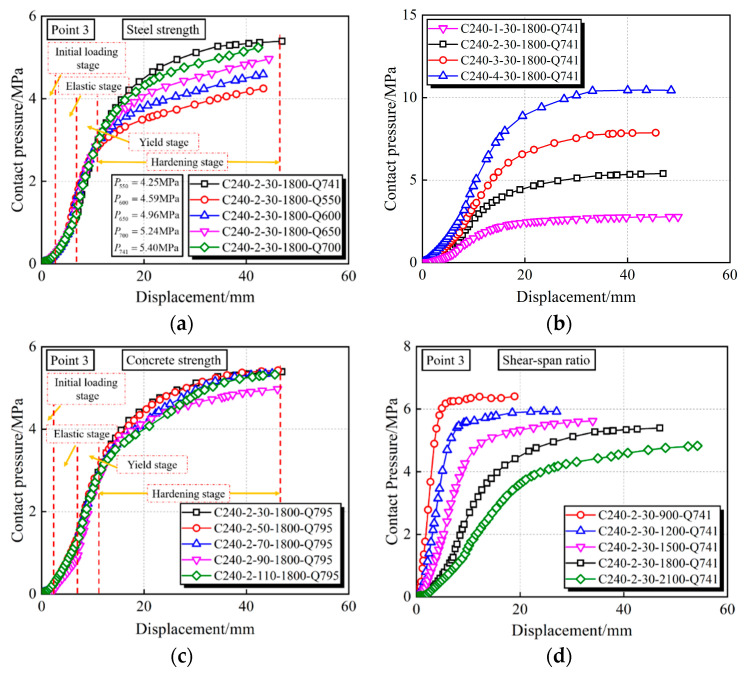
Influence on contact pressure. (**a**) Yield strength of steel tube; (**b**) *D*/*t* ratio; (**c**) Compressive strength of core concrete; (**d**) Shear-span ratio.

To examine the influence of various parameters on the mechanical properties of the CFHST beam, a parametric study was therefore conducted. Figure 14 shows the comprehensive effects of the tested parameters on bearing capacity and stiffness, including steel yield strength, *D*/*t* ratio, concrete strength and shear-span ratio. Figure 15 displays the influence on the full-range load–displacement curves. Figure 16 depicts the influence on the contact pressure at Point 3 between the steel tube and core concrete.

By increasing the steel yield strength from 550 to 741 MPa, the bearing capacity compared to the benchmark specimen C240-2-30-1800-Q550 can be, respectively, enhanced by 7%, 12%, 16% and 20%, as shown in Figure 14 and Figure 15a. Moreover, in Figure 16a, the contact pressure is increased by 8%, 17%, 23% and 27%, respectively; however, there is a slight effect on the stiffness, as displayed in Figure 14 and Figure 15a. The influence of *D*/*t* ratios from 60 to 240 were investigated by keeping the diameter constant. Compared with the maximum *D*/*t* ratio (*D*/*t* = 240), the vertical capacity of 60~120 is increased by 79%, 157%, and 235%, and the stiffness is increased by 103%, 158%, and 213%, respectively. This can be ascribed to a stronger confinement effect induced by increased wall thickness, therefore making an obvious enhancement to the interface contact pressure. Given the existing study, the interaction pressure of CFST members within *D*/*t* = 47~75 usually focuses on the scope of 2~5 MPa [36,37]; therefore, the CFHST members with out-of-code *D*/*t* ratios (e.g., *D*/*t* = 120 and 240) can perform reasonable composite behavior due to the acceptable contact pressure larger than 2.5 MPa. Using HS steel can provide a higher confinement effect to the core concrete for the purpose of reduced material consumption.

As shown in Figure 15c and Figure 16c, CFHST members with concrete strengths from ~30 to ~110 MPa display a slight difference on the full-range load–displacement curves and contact pressure, and so does to the bearing capacity and stiffness (Figure 14). This phenomenon is mainly caused by similar concrete tension strength making insufficient use of maximum compressive strength as the core concrete gradually cracks. 

To study the influence of shear-span ratio on the flexural capacity of a CFHST member, various shear-span distances ranging from ~900 to ~2100 mm were examined. As depicted in Figure 14 and Figure 15d, compared with the specimen C240-2-30-2100, the initial stiffness is, respectively, increased by 46%, 103%, 192% and 348% as the shear-span ratios decrease, and the vertical bearing capacity is increased by 31%, 63%, 104% and 190%, respectively. Through Figure 16d, the contact pressure significantly increases as the shear-span ratios decrease, which is ascribed to the enhanced initial stiffness making the ever-smaller bending failure or larger-shear failure a possibility.

## 5. Calculation Method on Flexural Bearing Capacity

### 5.1. Establishment of Calculation Method

**Figure 17:**
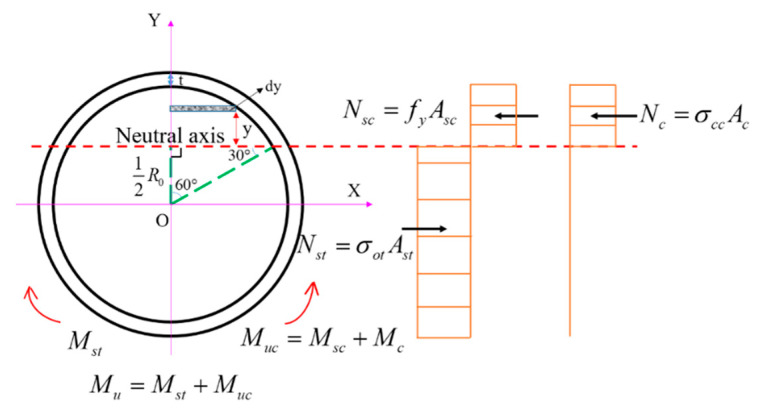
Stress distribution at the mid-span section.

A simplified plastic calculation method was used to determine maximum flexural capacity. Corresponding to the numerical and test analysis, the CFHST beam is considered to reach the ultimate state as the height of the neutral axis is distributed at the center line of the core concrete, as shown in Figure 17. Based on the following hypotheses, this calculation method can be established:

(1) The cross-section remains plane after deformation;
(2) The concrete in the tension zone is neglected for calculating the flexural capacity;
(3) The steel tube in the compression zone enters the yielding stage.

Therefore, according to the mechanical equilibrium, the following equation can be obtained.

(9) $$N_{c}+N_{sc}+N_{st}=0$$

(10) $$N_{c}=\sigma_{cc}A_{c}$$

(11) $$N_{sc}=f_{y}A_{sc}$$

(12) $$N_{st}=\sigma_{ot}A_{st}$$

(13) $$\sigma_{ot}=\left\{ \begin{array}{cc} \frac{N_{ct}+N_{sc}}{A_{c}} & \sigma_{ot}\leq f_{y}\\ f_{y} & \sigma_{ot}>f_{y} \end{array}\right.$$

where *N_c_* is the capacity contribution of the core concrete; *N_sc_* and *N_st_* are, respectively, the capacity contributions to compression and tension of HS steel tube; *A_c_*, *A_sc_*, and *A_st_* are, respectively, the compression area of concrete, the compression area and tension area of steel tube; and $\sigma_{ot}$ is the equivalent tensile stress of steel tube.

The compressive strength of the core concrete can be used by Sakino [38].

(14) $$\sigma_{cc}=\left\{ \begin{array}{cc} f_{c}+0.78\frac{2t}{D-2t}f_{y} & \sigma_{ot}\leq f_{y}\\ \frac{N_{st}-N_{sc}}{A_{c}} & \sigma_{ot}>f_{y} \end{array}\right.$$

where $\sigma_{cc}$ is the compressive strength of core concrete.

Then, the flexural bearing capacity can be obtained by superposing the moment of tensile stress and compressive stress:

(15) $$M_{u}=M_{uc}+M_{st}$$

(16) $$M_{uc}=M_{sc}+M_{c}$$

(17) $$M_{c}=2\cdot \sigma_{cc}\int_{\frac{1}{2}R_{0}}^{R_{0}}(y+R_{0}+t)\cdot \sqrt{R_{0}^{2}-y^{2}}dy$$

(18) $$M_{sc}=2\cdot f_{y}\cdot \int_{\frac{1}{2}R_{0}}^{R_{0}+t}(y+R_{0}+t)\cdot (\sqrt{{\left(R_{0}+t\right)}^{2}-y^{2}}-\sqrt{R_{0}{}^{2}-y^{2}})dy$$

(19) $$M_{st}=2\cdot \sigma_{ot}\cdot \int_{-(R_{0}+t)}^{\frac{1}{2}R_{0}}(y+R_{0}+t)\cdot (\sqrt{{\left(R_{0}+t\right)}^{2}-y^{2}}-\sqrt{R_{0}{}^{2}-y^{2}})dy$$

where *M_u_* is the total flexural capacity of CFHST members; *M_uc_* is the flexural capacity of steel tube and concrete in compression zone; *M_st_* is the bearing capacity of the steel tube in the tension zone; *M_sc_* and *M_c_* are the flexural contributions of the steel tube and concrete in compression zone, respectively; *y* is the distance away from the neutral axis; *R*_0_ is the radius of core concrete; and *t* is the thickness of steel tube.

### 5.2. Verification of Accuracy of Calculation Method

**Figure 18:**
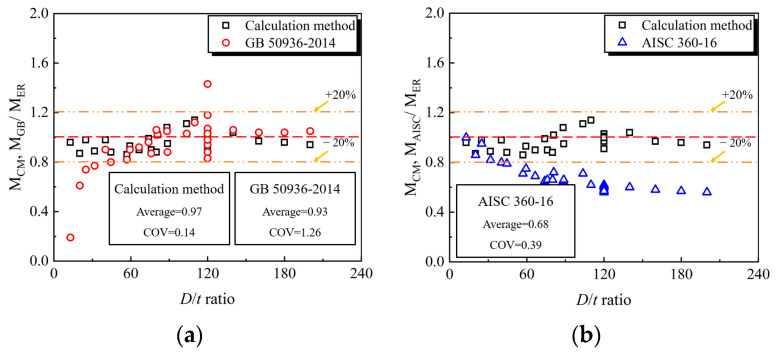

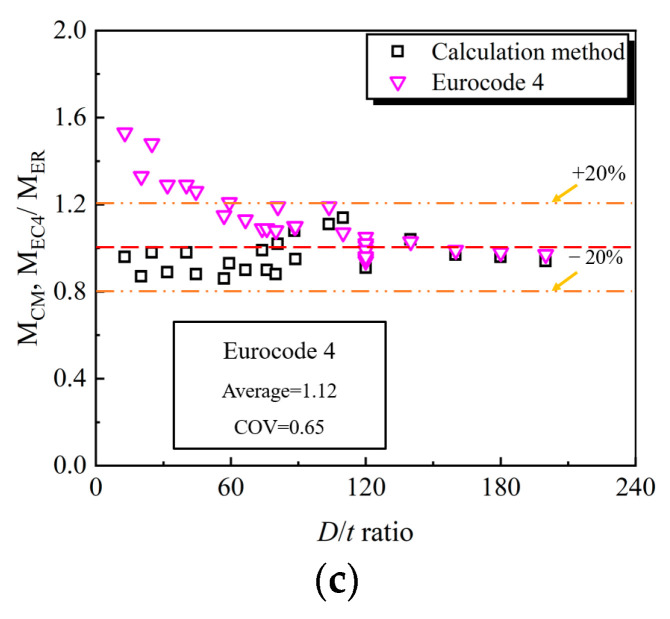
Comparison on prediction results of various methods. (**a**) GB 50936-2014; (**b**) AISC 360-16; (**c**) Eurocode 4.

To verify the accuracy of the proposed calculation method, the results of calculation method and related literature (e.g., the GB 50936-2014, AISC 360-16, and Eurocode 4) results are listed in Table 3 and Figure 18, where the method specified in AISC 360-16 performs a more conservative prediction trend compared to the counterparts in the codes of GB 50936-2014 and Eurocode 4.

Meanwhile, the simplified method in this paper achieves a reasonable accuracy in terms of the mean value (MV) of 0.97 compared to the corresponding MVs of 0.93, 0.68 and 1.12 of existing design codes. The differences in predicting ultimate bearing capacity may be ascribed to the different definitions in the ultimate limit state. Generally, the simplified method proposed in this paper can be accepted to perform structural design and performance evaluation, and pursuing a more accurate strength model is also the continued aim.

## 6. Conclusions

This paper systematically investigated the flexural behavior of out-of-code CFHST members. Based on the test, FE and theoretical analysis, some valuable conclusions can be drawn within the scope of current research:

1. The tested CFHST members fail in a mode of steel fracture and concrete cracks in the tension zone, as well as local buckling in the compression zone, to form a plastic hinge with ductile behavior. Due to the higher confinement effect of HS steel, the strain distribution along the section height approximately adopts a linear trend, revealing that the plane cross-section assumption is still justified for CFHST members with out-of-code *D*/*t* ratios.
2. The FE model was developed and verified for the analysis of full-range composite behavior; meanwhile, the load–displacement curves can be divided into three various working stages. The HS thin-walled steel tube experiences an arch mechanism similar to the core concrete, where the maximum tensile stress approximately transmits in an angle of 45° and vice versa.
3. A detailed parametric study was conducted to examine the influences of key parameters (e.g., the material strength, *D*/*t* ratio and shear-span ratio) on the full-range load–displacement curves and interaction behavior, in which the CFHST members with out-of-code *D*/*t* ratios (e.g., *D*/*t* = 120 and 240) can perform with reasonable composite behavior due to an acceptable contact pressure larger than 2.5 MPa.
4. A simplified theoretical model for calculating flexural capacity was derived and established by achieving a MV of 0.97 and a COV of 0.14 compared to the prediction results of existing design codes. Meanwhile, the method specified in AISC 360-16 performs a more conservative prediction trend compared to the counterparts in the codes of GB 50936-2014 and Eurocode 4. Therefore, the simplified method in this paper can be accepted as a primary tool to conduct structural design and performance evaluation.

In this study, the influence of shear force and shear-span ratio could not be completely excluded in the test. Although the bending moment plays a leading role in the bearing capacity of specimens with a large shear-span ratio and the shear force has little influence on the bearing capacity, the influence of shear force on the flexural bearing capacity could not be completely excluded. In future research, a four-point bending test should be considered, and the diameter–thickness ratio and concrete strength should be studied while keeping the diameters of specimens unchanged.

## Figures and Tables

**Table 1:** Designed parameters of CFHST beams.

Group	Specimens	*D*/mm	*t*/mm	*L*/mm	*D*/*t* Ratios	Steel Grade	Concrete Grade
C240	C240-1	240.8	1.98	1800	121.62	Q690	C50
	C240-2	241.24	2.15	1800	112.2		
	C240-3	241.4	1.97	1800	122.54		
C280	C280-1	281.39	2.05	1800	137.26	Q690	C50
	C280-2	280.83	2.06	1800	136.33		
	C280-3	281.74	2.1	1800	134.16		
C320	C320-1	320.52	2.08	1800	154.1	Q690	C50
	C320-2	320.91	2.03	1800	158.08		
	C320-3	320.3	2.04	1800	157.01		
C360	C360-1	360.33	2.03	1800	177.5	Q690	C50
	C360-2	360.88	2.01	1800	179.54		
	C360-3	360.34	1.96	1800	183.85		
C400	C400-1	400.51	2.19	1800	182.88	Q690	C50
	C400-2	400.85	2.29	1800	175.04		
	C400-3	400.22	1.99	1800	201.12		

Note: *D*, *t* and *L* are the measured diameter, steel tube thickness and span length of CFHST members, respectively.

**Table 2:** Mix proportion of concrete.

Concrete Grade	Weight/[kg/m^3^]					
	Fly Ash	Water	Cement	Sand	Gravel	Water Reducer
C50/60	80	170	360	693	1084	13.2

**Table 3:** Comparison results between the experimental/FE results and design methods.

No.	Specimen	*f_y_*/MPa	*D*/*t*	Test/FE Results (kN·m)	Proposed Method		GB 50936-2014		AISC 360-16		Eurocode 4		Ref.
					Results (kN·m)	C/R	Results (kN·m)	G/R	Results (kN·m)	A/R	Results (kN·m)	E/R	
1	CBC0-C	400	109.9	7.6	8.65	1.14	8.48	1.12	4.74	0.62	8.11	1.07	[39]
2	CBC0-B	400	88.32	9.1	9.82	1.08	9.51	1.05	5.96	0.65	10.03	1.10	
3	CBC0-A	400	73.93	11	10.84	0.99	10.53	0.96	7.18	0.65	11.95	1.09	
4	CBC1	365	40.25	11.33	11.13	0.98	10.19	0.90	9.11	0.80	14.66	1.29	
5	CBC2	432	31.77	10.86	9.66	0.89	8.32	0.77	8.89	0.82	14.03	1.29	
6	CBC5	433	24.86	3.78	3.70	0.98	2.81	0.74	3.58	0.95	5.59	1.48	
7	CBC7	408	20.16	4.75	4.13	0.87	2.90	0.61	4.09	0.86	6.33	1.33	
8	CBC9	460	12.84	1.17	1.13	0.96	0.23	0.19	1.18	1.00	1.79	1.53	
9	C133X1.5C30	465	88.67	18.12	17.14	0.95	15.87	0.88	11.98	0.66	19.87	1.10	[40]
10	C133X2.0C30	436	66.5	21.54	19.45	0.90	19.76	0.92	14.97	0.69	24.45	1.13	
11	C114X1.1C30	448	103.6	8.85	9.81	1.11	9.13	1.03	6.28	0.71	10.54	1.19	
12	C114X1.5C30	444	76	12.69	11.48	0.90	11.04	0.87	8.43	0.66	13.87	1.09	
13	C114X2.0C30	458	57	16.17	13.88	0.86	13.34	0.82	11.49	0.71	18.56	1.15	
14	C89X1.1C30	434	80.90	5.11	5.21	1.02	5.24	1.02	3.69	0.72	6.10	1.19	
15	C89X1.5C30	447	59.33	6.85	6.34	0.93	6.14	0.90	5.13	0.75	8.32	1.21	
16	C89X2.0C30	474	44.5	9.07	7.97	0.88	7.25	0.80	7.18	0.79	11.42	1.26	
17	C240-2-30-1800	741	120	134.89	136.20	1.00	123.96	1.07	83.95	0.62	141.38	1.05	[–]
18	C280-2-30-1800	741	140	189.72	197.77	1.04	183.31	1.06	114.54	0.60	194.87	1.03	
19	C320-2-30-1800	741	160	259.91	253.12	0.97	258.08	1.04	149.87	0.58	257.26	0.99	
20	C360-2-30-1800	741	180	335.66	320.58	0.96	349.92	1.04	189.94	0.57	328.67	0.98	
21	C400-2-30-1800	741	200	421.48	395.99	0.94	460.48	1.05	234.76	0.56	409.18	0.97	
22	C240-3-30-1800	741	80	193.78	169.63	0.88	154.87	1.06	126.99	0.66	208.58	1.08	
23	C240-2-50-1800	741	120	138.26	142.09	1.03	162.94	1.18	83.95	0.61	145.54	1.05	
24	C240-2-70-1800	741	120	141	142.09	1.01	202.20	1.43	83.95	0.60	148.40	1.05	
25	C240-2-30-1500	741	120	140.33	136.20	0.97	123.96	0.88	83.95	0.60	141.38	1.01	
26	C240-2-30-2100	741	120	149.93	136.20	0.91	123.96	0.83	83.95	0.56	141.38	0.94	
27	C240-2-30-1200	741	120	148.88	136.20	0.91	123.96	0.83	83.95	0.56	141.38	0.95	
28	C240-2-30-1800-Q700	700	120	131.18	131.55	1.00	123.96	0.94	79.30	0.60	133.99	1.02	
29	C240-2-30-1800-Q650	650	120	125.98	125.87	1.00	123.96	0.98	73.64	0.58	124.94	0.99	
30	C240-2-30-1800-Q600	600	120	120.06	120.20	1.00	123.96	1.03	67.97	0.57	115.85	0.96	
MV						0.97		0.93		0.68		1.12	
COV						0.14		1.26		0.39		0.65	

Note: MV indicates the mean value; COV means the covariance. R represents the benchmark results of test or FE models; C, G, A and E, respectively, denote the results of the proposed calculation method, GB 50936-2014, AISC 360-16 and Eurocode 4.

## Data Availability

Not applicable.
